# Switching Cytolytic Nanopores into Antimicrobial Fractal Ruptures by a Single Side Chain Mutation

**DOI:** 10.1021/acsnano.1c00218

**Published:** 2021-04-22

**Authors:** Katharine Hammond, Flaviu Cipcigan, Kareem Al Nahas, Valeria Losasso, Helen Lewis, Jehangir Cama, Fausto Martelli, Patrick W Simcock, Marcus Fletcher, Jascindra Ravi, Phillip J Stansfeld, Stefano Pagliara, Bart W Hoogenboom, Ulrich F Keyser, Mark S P Sansom, Jason Crain, Maxim G Ryadnov

**Affiliations:** 1 National Physical Laboratory, Hampton Road, Teddington, TW11 0LW, UK; 2 London Centre for Nanotechnology, University College London, London WC1H 0AH, UK; 3 Department of Physics & Astronomy, University College London, London WC1E 6BT, UK; 4 IBM Research Europe, Hartree Centre, Daresbury WA4 4AD, UK; 5 Cavendish Laboratory, University of Cambridge, Cambridge CB3 0HE, UK; 6 STFC Daresbury Laboratories, Daresbury WA4 4AD, UK; 7 Living Systems Institute, University of Exeter, Exeter EX4 4QD, UK; 8 College of Engineering, Mathematics and Phys Sciences, University of Exeter, Exeter EX4 4QF, UK; 9 Department of Biochemistry, University of Oxford, Oxford OX1 3QU, UK; 10 College of Life and Environmental Sciences, University of Exeter, Exeter EX4 4QD, UK; 11 Department of Physics, King’s College London, London, WC2R 2LS, UK

**Keywords:** innate host defense, antibiotics, nanopores, *de novo* protein design, nanoscale imaging

## Abstract

Disruption of cell membranes is a fundamental host defence response found in virtually all forms of life. The molecular mechanisms vary but generally lead to energetically favored circular nanopores. Here we report an elaborate fractal rupture pattern induced by a single side-chain mutation in ultrashort (8-11-mers) helical peptides, which otherwise form transmembrane pores. In contrast to known mechanisms, this mode of membrane disruption is restricted to the upper leaflet of the bilayer where it exhibits propagating fronts of peptide-lipid interfaces that are strikingly similar to viscous instabilities in fluid flow. The two distinct disruption modes, pores and fractal patterns, are both strongly antimicrobial but only the fractal rupture is non-hemolytic. The results offer wide implications for elucidating differential membrane targeting phenomena defined at the nanoscale.

As a structural foundation of cellular membranes, phospholipid bilayers compartmentalise and regulate physical and biological processes at the nanoscale.^1^ While their integrity is fundamental for cell survival, the bilayers undergo different forms of disruption induced by processes as diverse as endo- and exocytosis, membrane fusion and repair.^2–4^ The disruption is associated with the formation of nanoscale pores that can be transient, expanding or irreversible. Bacteria form pores in host cells to access nutrients or porate other bacteria competing for the same ecological niches, while multicellular organisms employ poration to target microbial membranes or activate intrinsic suicide programs in aberrant cells.^5–8^ Pores can be heterogeneous or conserved in size and form as transmembrane channels or monolayer pits.^7–9^ Despite their rich variety and origin, all pores appear to be circular.

The circular geometry is consistent with the classical continuum mechanics model of a membrane as a thin featureless elastic sheet under lateral tension (*σ*).^10–13^ Pore formation occurs as a result of two competing effects: a pore reduces energy but incurs a positive edge or line tension (*γ*) to maintain a given perimeter. These considerations imply that circular pores of radius (*r*) (enclosing the largest area of the perimeter) are energetically favoured over any other arbitrary geometry with an energy (relative to the intact membrane) defined by

(1) $$E=2\pi ry-\pi r^{2}\sigma$$

which yields a barrier for pore formation

(2) $$E^{*}=\frac{\pi \gamma^{2}}{\sigma }$$

and a critical radius

(3) $$r_{c}=\gamma /\sigma$$

Consequently, thermal fluctuations produce pores spontaneously which either reseal for *r* < *r_c_* or expand indefinitely for *r* > *r_c_*. However, this model neglects the consequences of growth dynamics and two-dimensional viscous flow of the membrane and does not apply to situations where membrane disruption is confined to individual leaflets. In such circumstances line tension and the nature of the exposed surface are significantly modified from the transmembrane channel case. Existing evidence supports the formation of monolayer or semi pores as a result,^9, 13–16^ while most recent computational models begin exploring metastable pre-pores in tension-free lipid bilayers.^17^

Here we show that a single side-chain mutation in ultra-short helical peptides (8-11 residues in length) switches classical transmembrane pores into fractal rupture patterns in the upper leaflet of the bilayer. We first discuss the principal finding that this mutation induces elaborately structured nanoscale patterns, which are unlike the known forms of peptide- and protein-membrane morphologies arising from phase separation. We then demonstrate biological implications for such a switch and explore the molecular origins of the mutation using computer simulations and molecular biophysics.

## Results and Discussion

### Design Rationale and Nanoscale Imaging

**Figure 1:**
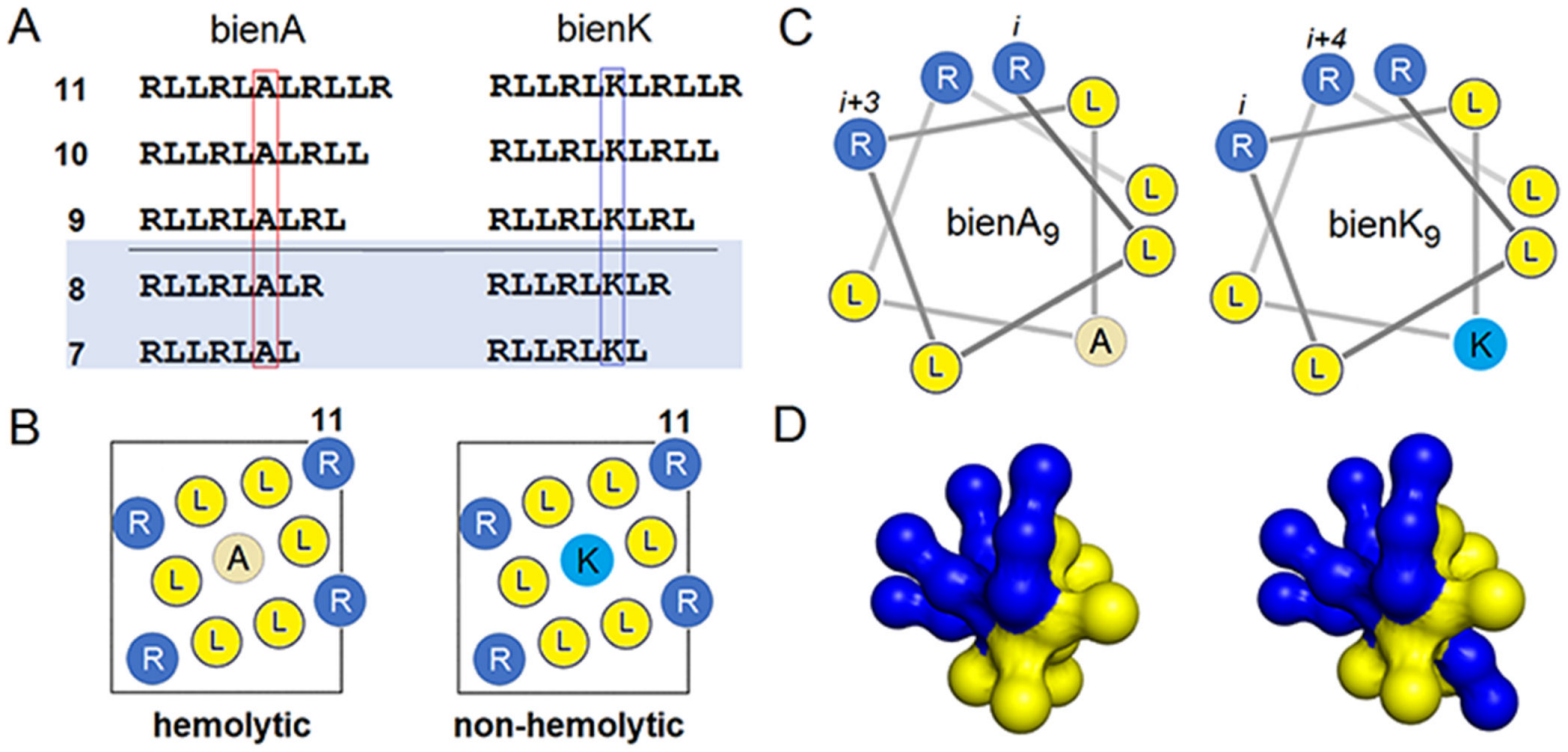
Structural motifs used in the study RLLRL*X*LRLLR, where *X* is A for bienA and K for bienK peptide series. (A) Linear amino acid sequences and configured onto (B) helical nets and (C) helical wheels with 3.6 residues per turn. Helical wheels for 9-mers are given for each series to exemplify the nomenclature used: bienA_9_ and bienK_9_. Helical spacings, *i, i+3* and *i, i+4* in the sequences place the same residues next to each other along the helical axis and help maintain interfacial contacts facilitating peptide assembly in membranes.^15^ Leucine provides strong hydrophobic interactions with the bilayer. Arginine has the strongest affinity to anionic lipids. The two amino acids have the same helical propensity. The blue box in (A) indicates biologically inactive sequences. Lysine and alanine residues are shown in light blue and yellow to help visually distinguish from the other residues. (D) Spatial coarse-grained models of bienA (left) and bienK (right).

We consider a minimal amphipathic helix with *bi*nary *en*coding (bien) by arginine and leucine, which form cationic and hydrophobic faces of the helix, respectively (Fig 1). Our design rationale builds upon the structure-function relationships of α-helical host defence peptides.^18–21^ Upon binding to membranes the peptides fold into amphipathic helices, in which polar and hydrophobic amino-acid side chains are partitioned into polar and hydrophobic faces, respectively.^20–22^ The peptides are typically cationic and favor anionic microbial membranes.^18, 22^ The ratio of hydrophobic-to-cationic residues in antimicrobial sequences can be used to guide empirical correlations with cytotoxicity.^23^ As a rule of thumb, ratios greater than 1 enhance the contribution of hydrophobic interactions to membrane binding.^21–24^ These interactions do not discriminate between microbial and mammalian membranes, and when increasing lead to cytolytic effects including hemolysis.^23, 24^ Similarly, longer sequences form more extensive cationic and hydrophobic faces, which support stronger membrane binding resulting in more profound and less selective antimicrobial activities.^18, 25^ To date, the role of physicochemical and structural properties of antimicrobial peptides in defining membrane disruption pathways have been studied using model and naturally occurring sequences, demonstrating good agreements between experimental evidence and computational descriptions.^18–28^ These studies primarily focus on elucidating membrane disruption pathways caused by particular peptides without attempting to provide a unifying mechanism between circular pore formation and possible non-porating phenomena.

Our study introduces such a mechanism using *de novo* sequences of minimal length and composition, which enable the formation of contiguous and perfectly amphipathic helices. Given the postulated requirement of at least eight or more residues for a sequence to form a helix,^29^ which constitutes a minimum length to arrange repeated hydrogen bonding spacings (*i, i+4*), our helix design does not exceed three helical turns in length. This is done for two reasons. Firstly, these helices are not long enough to fold autonomously and must rely on binding to membranes to form. Secondly, the switched polarity of a single side chain in a relatively short hydrophobic face is deemed significant enough to alter the insertion mode of the helix and consequently its membrane disruption pathway – a conjecture that may find support in the literature reporting the impact of switching polarity in antimicrobial peptides on cell selectivity.^30–32^

Consistent with this reasoning, a single side-chain mutation was introduced in the hydrophobic face of the helix where it was computationally predicted to influence peptide insertion in the membrane (Figs S1, S2).^33^ Specifically, an alanine in the mutation position (bienA) was found to favor an insertion of the helix deep into the bilayer interface thus supporting transmembrane orientation and pore formation. In contrast, a lysine in this position (bienK) was found to favor a shallow insertion of the helix confining it to the upper (distal) leaflet of the bilayer (Figs 1 & S1, S2), in a manner similar to monolayer poration phenomena.^9, 14, 16^ To demonstrate the switch, a series of peptides of decreasing length was synthesized for bienA and bienK each to probe a length cut-off at which distinctive pathways were still apparent (Figs 1 & S3).

**Figure 2:**
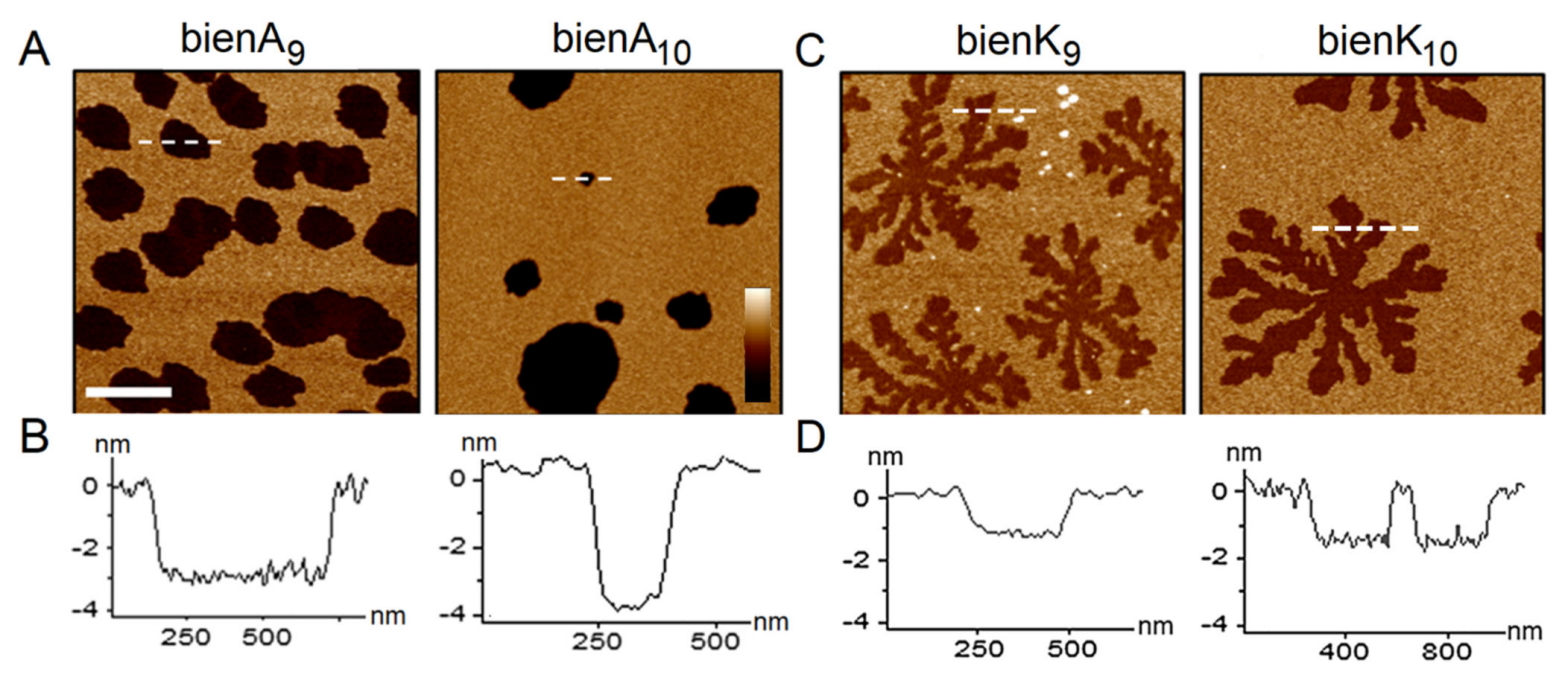
Membrane instability mode switching by a single side-chain mutation. In-liquid AFM images of anionic SLBs (DLPC/DLPG, 3:1 molar ratio) treated with (A) bienA and (C) bienK (0.3 μM peptide) over 20 min at room temperature. Length and height scale bars are 1 μm and 7 nm, respectively. (B) and (D) height profiles taken along the highlighted lines in (A) and (B), respectively, showing the depth of topography defects in the bilayers.

Membrane disruption was monitored directly using atomic force microscopy (AFM) performed in aqueous environments on supported lipid bilayers (SLBs), which were prepared by the surface deposition of unilamellar vesicles on appropriate substrates using established procedures.^34,35^ Anionic and zwitterionic vesicles were used to mimic microbial and mammalian membranes, respectively: 1,2-dilauroylphosphatidylcholine (DLPC) was used for zwitterionic vesicles, while its mixture with 1,2-dilauroyl-sn-glycero-3-phospho-(1′-rac-glycerol) (DLPG) at a 3:1 molar ratio for anionic vesicles.^7, 36–39^ SLBs provide flat surfaces (to within a few Å) in the unperturbed state, which allows accurate depth measurements of membrane defects caused by peptide treatment. For bienA series the formation of conventional transmembrane pores was apparent in anionic SLBs. The pores appeared circular in shape (Fig 2A, B), with morphologies similar to circular pores reported for other peptides (Fig S4),^21,28,40,41^ and tended to expand and merge with larger defects observed for longer sequences (Figs S5 & S6).

The single side-chain mutation (A→K) resulted in a radically different disruption pathway. For the bienK series membrane defects in anionic SLBs adopted a floral or fractal growth pattern (Fig 2C). Fractal dimensions marginally increased from the order of 1.73 for bienK_9_ to 1.91 for bienK_11_, retaining the fractal pattern (Fig S7). The depths of these patterns were at half the thickness of the bilayer, which is indicative of that the lipid disruption was confined to the distal leaflet of the bilayer without forming a transmembrane channel (Figs 2D, S5).^9^ Distinctive was also the behaviour of each series in zwitterionic SLBs: bienA formed small transmembrane pores in the bilayers, whereas no defects were observed for bienK (Fig S8). Thus, the results indicate that the single side chain mutation in the hydrophobic face of the helix controls the pathway of membrane disruption, from transmembrane poration to fractal rupture patterns, while being selective towards negatively charged phospholipid bilayers.

### Biological Activity and Differentiation

Irrespective of variations in the topography of membrane ruptures, the defects in both series were evident for sequences comprising more than eight residues. The length of eight residues was found to be the cut-off for biological activity (Fig 1A, Table S1). Sequences comprising eight and seven residues were biologically inactive (Table S1). This can be attributed to that sequences shorter than nine residues form weak helices,^29^ which could not inflict appreciable damage to phospholipid bilayers or lyse bacterial cells. For 9-mers and longer sequences minimum inhibitory concentrations (MICs) were found to be comparable with MICs of antimicrobial peptides and antibiotics (Table S1).

Notably, in contrast to bienA, which exhibited significant hemolytic activities, the bienK peptides were non-hemolytic (Table S1). This differential biological activity of bienK peptides correlated well with their selectivity towards bacterial membranes as gauged by AFM (Fig S8). For each biologically active peptide of the series the ratio of hydrophobic-to-cationic residues is >1. In membrane-active peptides this ratio supports the formation of more extensive hydrophobic faces characteristic of hemolytic and venomous peptides such as melittin (Table S1).^21–24^ For bienK this is not the case since the lysyl side chain switches the polarity of the hydrophobic face thereby splitting it into two smaller hydrophobic subfaces. This switch changes the membrane disruption pattern from hemolytic to non-hemolytic, which is consistent with observations by others.^30–32^ These findings suggest that bienA peptides cause irreversible damage to membranes acting as non-selective membranolytic agents. By contrast, cell membranes may better recover from the fractal ruptures of bienK peptides whose selectivity towards bacterial cells may involve complementary killing routes, such as metabolic inhibition.^42^

**Figure 3:**
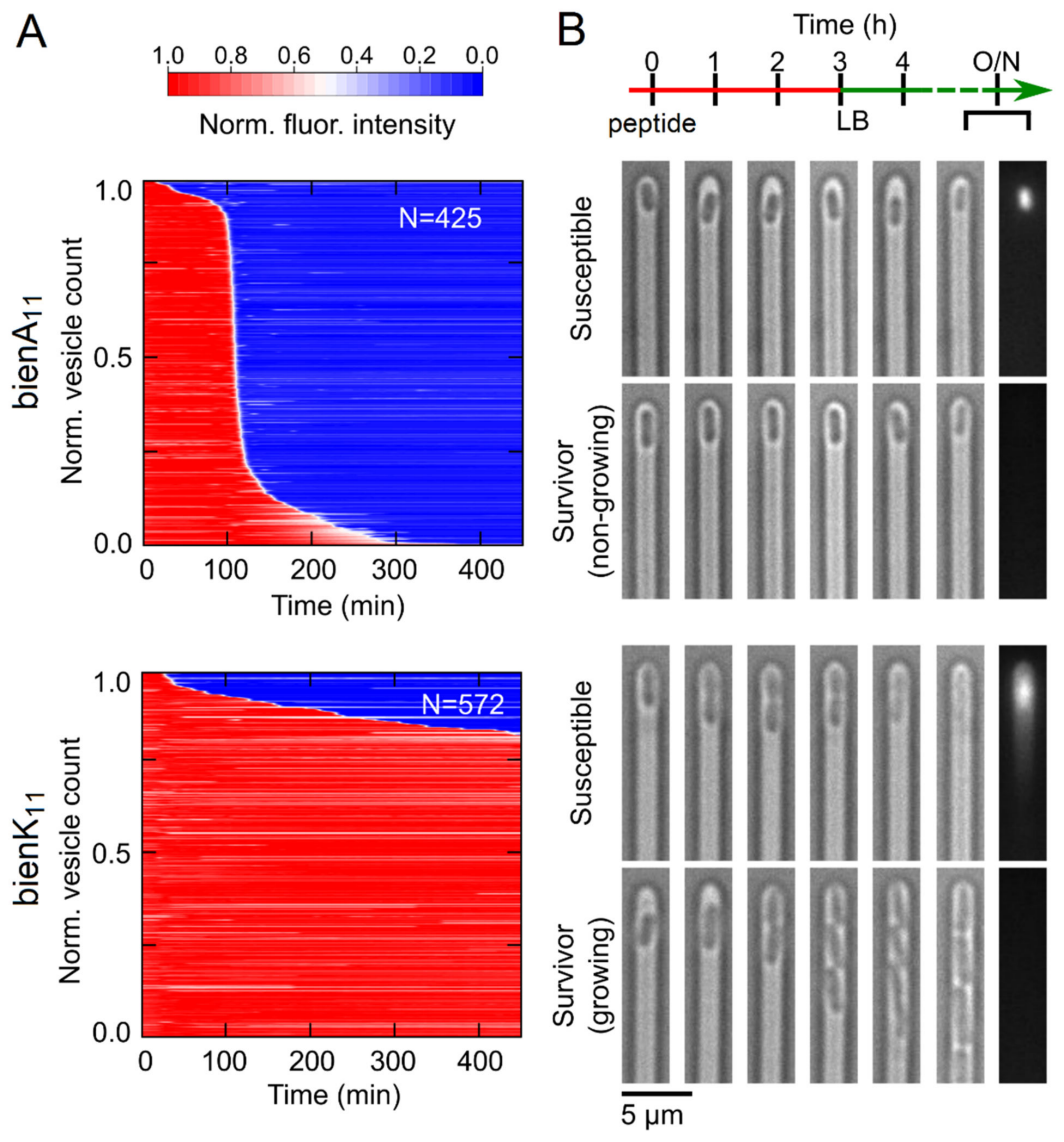
Functional implications of the two rupture pathways. (A) Summaries of membranolytic activities for bienA_11_ and bienK_11_ (10 μM peptide) against anionic GUVs (DOPC/DOPG, 3:1 molar ratio). Each horizontal line depicts the normalised intensity of an encapsulated dye (fluorescent dye 8-hydroxypyrene-1,3,6-trisulfonic acid) in a single trapped vesicle over time, after background subtraction. GUV membranes, intact and compromised, are at high (red) and low (blue) fluorescence intensities, respectively. N denotes the number of analysed vesicles. (B) Two panels of optical micrographs showing individual microfluidic channels with *E. coli* cells during peptide treatment: 10 μM peptide is added (0 hours) and then bright-field micrographs were taken at hourly intervals. After 3 hours of incubation with peptide the cells were flushed with fresh lysogeny broth (LB) and the incubation was continued to determine cell re-growth at 4 hrs. After the following overnight incubation (O/N) the cells were stained with propidium iodide, which is a live–dead stain entering dead bacteria whose membranes are compromised. Fluorescence images of the O/N samples are the last micrographs (to the right) in the panels. Fluorescent cells are susceptible cells lysed by peptide. Non-fluorescent cells are survivor cells of two types: non-growing, non-dividing cells (upper panel) and cells that were dividing during the treatment (lower panel).

To probe this conjecture, dye release experiments were performed using a bespoke microfluidic assay on anionic giant unilamellar vesicles (GUVs), which were assembled from 1,2-dioleoyl-sn-glycero-3-phosphocholine (DOPC) and 1,2-dioleoyl-sn-glycero-3-phospho-(1’-rac-glycerol) (DOPG) at 3:1 molar ratio.^36–39, 43^ Membranolytic activity was evident for bienA peptides which caused complete lysis of GUV populations within 300 min post addition (Figs 3A & S9). Up to three quarters of vesicles could survive lysis by bienK even over prolonged treatments (Figs 3A & S9). The data within each series was comparable at micromolar concentrations, which were in the range of MICs obtained for the peptides, indicating that a threshold concentration was reached in this concentration range for both series (Fig S10). The results suggest that phospholipid membranes should have higher recovery rates against bienK or are less affected by it. This could be due to that bienK peptides do not distribute to both sides of the bilayer or due to resealing effects observed in GUVs treated with antimicrobial peptides.^44, 45^

Variations in MICs could also be ascertained. However, MICs provide endpoint results for treated cell populations taking no account of changes at sub-population or cellular levels. To relate MICs to single-cell kinetics within the timescale of SLB and GUV measurements, hundreds of bacterial cells (*E. coli*) were screened in a multi-channel microfluidic device. Each channel traps one cell allowing it to maintain its phenotypic inheritance over an infinite number of generations. This helps monitor antimicrobial kinetics and phenotypic responses *in situ*.^46^ In response to bienA, only a small sub-population of viable-but-non-culturable cells survived the treatment but failed to regrow, conforming to the membranolytic nature of bienA in GUVs at the same concentrations used (10 μM) (Fig 3B). Distinctively more heterogeneous were cell responses to bienK, consistent with the GUV responses. Susceptible cells tended to start elongating before growth arrest and death, while cells that resisted bienK were able to grow and divide during the treatment (Fig 3B).

### Molecular Model and Characterisation

**Figure 4:**
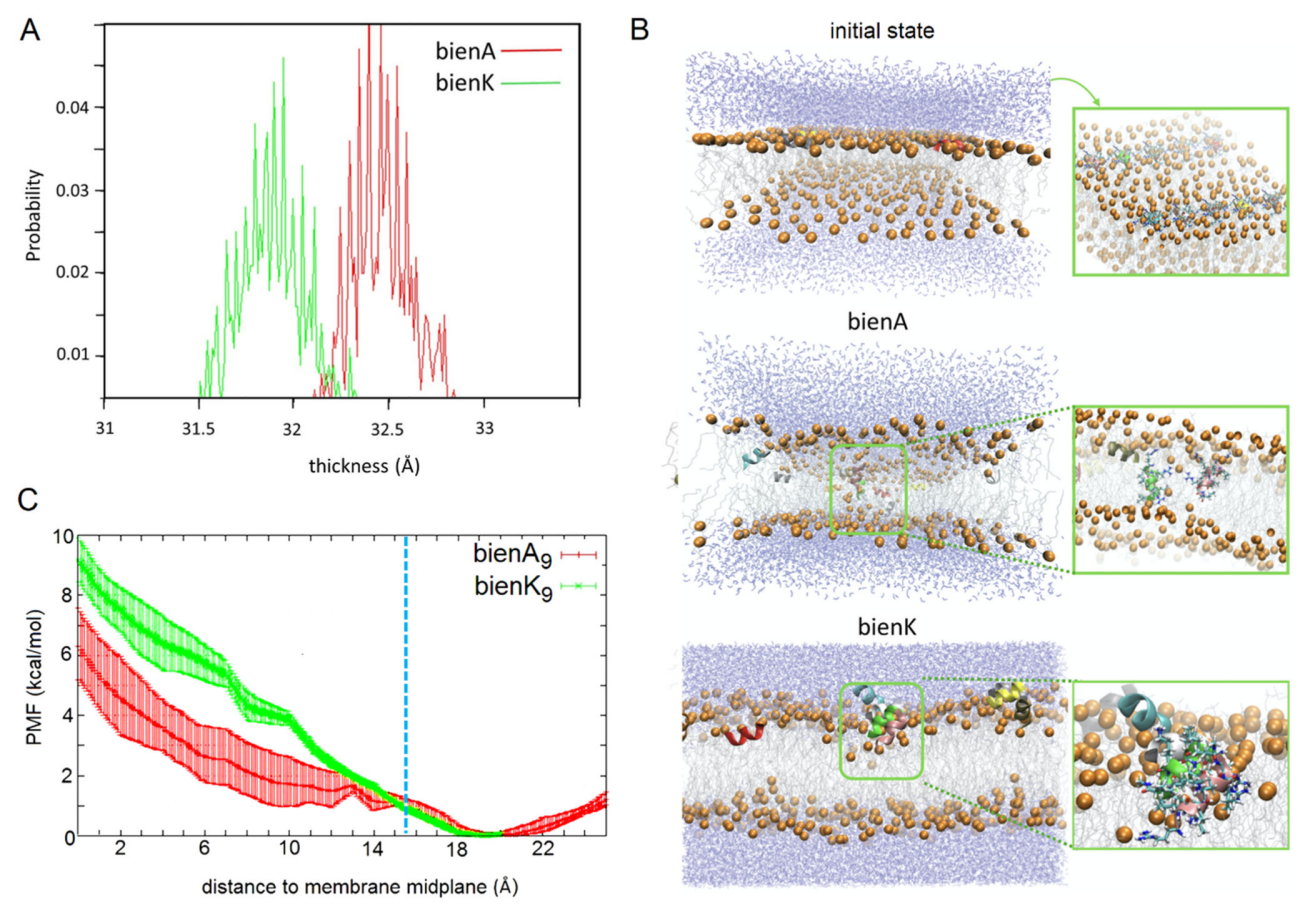
MD simulations of peptide insertion into phospholipid bilayers. (A) 1-μs simulations of bienA_9_ and beinK_9_ run for ten molecules each showing distributions of an average thickness of DLPC/DLPG (3:1 molar ratio) membranes. (B) Snapshots of MD simulations for the initial state and after 500 ns into simulations for each series (right panel) and more detailed snapshots highlighting peptide-lipid interactions (left panel). Key: blue lines denote water, orange spheres are phosphates, grey lines are lipid tails, cartoon and stick representations are used for peptides. (C) PMF over a distance to the membrane midplane for bienA_9_ (red) and protonated bienK_9_ (green). The dashed blue line denotes the membrane surface.

Consistent with the distinctive behaviours of the two series by AFM and biological tests, molecular dynamics (MD) simulations revealed that bienK strongly binds to phospholipid headgroups and pulls them towards the midplane interface compressing the bilayer (Fig 4A, B). By contrast, bienA increases the membrane area per lipid by escaping perpendicular orientation to membrane normal and favoring transmembrane insertion, leading to the formation of a water-filled transmembrane channel (Figs 4B, S11A-C). This is also reflected in a much higher probability of water molecules penetrating the bilayer for bienA (Figs S11D). The potential of mean force (PMF) measured over a distance to the membrane midplane proved to be significantly lower for bienA than for the protonated bienK (Fig 4C). This indicates that bienA remains deep in the bilayer facilitating poration, as was observed.

In bienK, lysine, unlike arginine, can be deprotonated in membranes,^47^ which allows it to fit in the hydrophobic bilayer interface. Upon protonation the lysyl side chain transforms into a cation, which introduces electrostatic repulsions between the hydrophobic faces of bienK molecules and competition with their cationic faces for phosphate groups.^47–49^ Therefore, the lysyl side chain re-orients towards phospholipid headgroups resulted in a shallower insertion of bienK (Figs 4B, S2). This differentiator should carry a cost of destabilizing the helix. Indeed, this was observed. Circular dichroism (CD) spectroscopy showed that bienK peptides were less helical than bienA in anionic unilamellar vesicles (Fig S12A, B). No folding was apparent for bienK in zwitterionic vesicles: CD spectra were indicative of random-coil conformations (Fig S12C). Unlike the lysine, the alanyl side chain could readily orient in the hydrophobic environment of the bilayer interface, which allows bienA to retain its hydrophobic face undisrupted thereby enhancing the contribution of the hydrophobic effect on folding.^50^ As a result, longer sequences of bienA retained helicity (Fig S12D).

Isothermal titration calorimetry (ITC) was used to gain a more quantitative insight into peptide-membrane interactions. For bienA_9_, peptide titrations into anionic phospholipid membranes gave an initial exothermic process revealing enthalpy-driven ionic and hydrogen-bond interactions (Fig S13A). With increasing peptide-lipid ratios, endothermic processes were more apparent suggesting an increase in hydrophobic surface area^51^ as a consequence of peptide insertion into the bilayer (Fig S13A). This process showed appreciable gain in binding free energy (ΔG of -7.2 kcal/mol) and dissociation constant (K_D_) of 0.17 μM. Characteristic of pore-forming antibiotics,^52^ these values reflect changes in bound-unbound water and its release from the apposed hydrophobic surfaces of peptides and the bilayer, which proves consistent with the results of MD simulations. By contrast, binding isotherms obtained for bienK_9_ gave a large K_D_ (2.12 μM) and a positive ΔG (1.03 kcal/mol), indicating an entropy-favoured process (Fig S13A). With no binding detected in zwitterionic vesicles for either of the series (Fig S13B), it can be concluded that the two modes of membrane disruption observed by AFM derive from two thermodynamically distinctive pathways of membrane binding and are reactant (peptide) driven.

As complex, multi-component systems, lipid membranes can adopt various forms of lateral segregation. Liquid nanoscale domains can be controlled by temperature and mechanical stimuli, while binary and ternary lipid mixtures can form liquid, gel or so-called ripple phases and exhibit broader behaviors including miscibility transitions, spinodal decomposition and viscous fingering in giant vesicles.^53–55^ These domains have distinct mechanical properties and interfacial tension at coexisting phase boundaries, which can lead to preferential segregation of proteins.^56, 57^

Most recently, pattern-forming morphologies in supported lipid bilayers were reported in the so-called double bilayers.^58, 59^ These morphologies bear relevance to our study as they exhibit similar growth dynamics and dimensions to the fractal ruptures caused by bienK in single bilayers. These morphologies are driven not by the switched polarity of adsorbed peptides, as in our case, but by tensile stress during flow.^59^ The rupture patterns are described to form in the distal bilayer of a double bilayer through burst events giving rise to branched defects.^58^ To describe these defects, equation (1) must include pore growth dynamics which obey a form of Stokes equation:

(4) $$\nabla \sigma =\xi \vec{\nu }-\mu \nabla^{2}\vec{\nu }$$

with viscosity (*μ*) and co-efficient (*ξ*) representing frictional interactions in lipid bilayers.^60^ From this a relaxation time $\tau_{r}=\left(\frac{\xi }{\gamma }\right)L$ can be derived for a pore perimeter (*L*). If membrane rupture occurs at a speed *ν* such that its propagation timescale, *τ_p_ = L/υ*, is faster than the membrane relaxation time, the pore edge response is too slow to establish the energetically favored circular periphery. Above critical length scale $L_{C}={\left(\frac{\gamma }{\xi \upsilon }\right)}^{1/2}$ irregular interfaces appear with morphologies like Staffman-Taylor instabilities in fluid flow as found by AFM for the bienK series (Fig 2C).

In double bilayers such instabilities lead to complete transmembrane ruptures formed spontaneously under tensile stress in the distal bilayer of a double bilayer.^58^ This is in contrast to the fractal ruptures in our study, which are caused by a prescribed chemical switch (Ala→Lys mutation) in the distal leaflet of a single bilayer. The dissipation caused in double bilayers is composed of sliding friction between the upper and lower bilayers, and the spreading of the lower bilayer on the surface. In bienK, the geometry is simpler. It is a single bilayer, in which non-trivial defects observed are confined to the upper leaflet. The relevant friction coefficient in this case is the inter-leaflet friction with the tension gradient provided by the surface-bound bienK. This case is significantly different from the double bilayer: in SLBs the lower leaflet has no net velocity. To rupture SLBs local tensile stress must be induced by chemical cues, bienK peptides in our case, which adhere to the surface of the bilayer, insert into it and assemble into peptide-lipid interfaces in the sites of insertion. The ruptures initiate rapidly into branching defects followed by fingering instabilities proceeding more slowly. Indeed, fractal ruptures formed in SLBs within minutes after adding bienK (Fig S14), evolving marginally over an hour (Movie S1). The ruptures remained conserved in morphology and depth over time, and unlike membrane thinning effects, which enhance pore formation,^44^ did not evolve into pores. The fractal dimensions of these ruptures proved to be peptide determined and decreased with decreasing peptide length (Fig S7). Fractal patterns could still be observed for bienK_8_ whose dimensions were comparable to those of bienK_11_ (Fig S5). In both these peptides the C-terminal residue is arginine, which provides five hydrogen-bond donors enhancing affinity towards phosphate groups.^47^ By trapping more phosphate and water, bienK_11_ promoted faster growth and lateral expansion (Fig S14).

To realize a practical model for these observations it may also be necessary to modify the application of equation (1) to account for the observation of anomalous disruption modes being restricted to the upper leaflet. In this case the estimates of surface and perimeter tensions are both significantly different from their transmembrane forms. Unlike the case of a transmembrane pore, disruption of the upper leaflet only exposes a hydrophobic surface to the surrounding water leading to an inverted situation where the “elastic sheet” model fails, and disruptions of large perimeter and smaller areas are preferred geometries. Further work is in progress to devise a realistic elastic model and simulation of such a situation.

## Conclusions

Our present results show that the switched polarity of a single side chain in the hydrophobic face of amphipathic peptides activates peptide-lipid interfaces causing a rupture pathway that appears distinct from known manifestations of membrane heterogeneities, phase separation, pore formation phenomena or other forms of lateral segregation. These chemically induced interfaces can be considered as charged equipotential surfaces having a degree of translational freedom that circular edges are too slow and sterically hindering to accommodate. Peptides in these interfaces adopt shallow insertion geometries that can arrange an aperture only in the upper distal leaflet. This exposes the hydrophobic proximal layer of membranes resulting in unfavorable water-lipid interfaces, which creates a situation that fundamentally changes the assumptions built into equation (1). The preferred geometry is no longer circular.^58–61^ Instead, structured defects with large perimeters and small areas are formed.

Molecular mechanisms of various pore-forming and membrane disruption phenomena found in peptides, proteins, antibiotics and various nanoparticles have been intensively studied using supported lipid bilayers.^62–66^ However, a strategy to elucidate a unifying mechanism has not emerged. The present study used a relatively simple strategy, which allowed to reveal a physical mode of membrane re-modelling, which to our knowledge has neither been previously observed nor proposed. The sensitivity of the chemical (peptide) triggers that separate conventional membrane poration from anomalous membrane disruption pathways is particularly striking. The two mechanisms are closely competing processes, which are independently activated by a single residue mutation. Computed potentials of mean force and simulations at the molecular scale give an early insight into insertion energetics and structural bias with resolutions capable of discriminating between single residue mutations. Future work on peptide–lipid organisation in the fractal pathways of membrane disruption and the nature of the exposed surfaces will be required to realize a refined theoretical model which includes the essential physics of these modes either at molecular or coarse-grained length scales. Extending and developing the notion of a minimal switch based on first design principles but which select between discrete disruption pathways in reconstituted bilayer systems offer the basis of a useful and systematic approach to devising more unified models of membrane instabilities.

## Methods

### Peptide synthesis

All peptides used in the study were made using a Liberty microwave peptide synthesizer (CEM Corp.) Conventional Fmoc/tBu solid-phase protocols and HBTU/DIPEA as coupling reagents were used. Rink amide 4-methylbenzhydrylamine resin was used for all peptides. After cleavage and deprotection (95% TFA, 2.5% TIS, 2.5% water) peptides were identified by analytical RP-HPLC and MALDI-ToF mass spectrometry.

MS [M+H]^+^: bienA_7_ – *m/z* 853.1 (calc), 855.6 (observed); bienA_8_ – *m/z* 1009.3 (calc), 1011.6 (observed); bienA_9_ – *m/z* 1122.4 (calc), 1125.0 (observed); bienA_10_ – *m/z* 1235.6 (calc), 1238.4 (observed); bienA_11_ – *m/z* 1391.8 (calc), 1395.6 (observed); bienK_7_ – *m/z* 910.2 (calc), 910.8 (observed); bienK_8_ – *m/z* 1066.4 (calc), 1069.9 (observed); bienK_9_ – *m/z* 1179.5 (calc), 1182.2 (observed); bienK_10_ – *m/z* 1292.7 (calc), 1295.6 (observed); bienK_11_ – *m/z* 1448.9 (calc), 1451.2 (observed); melittin – *m/z* 2846.5 (calc), 2846.7 (observed).

### Reversed-phase high performance liquid chromatography

A Thermo Scientific Dionex RP-HPLC system (Ultimate 3000) was used to purify and analyse the peptides. Vydac C18 analytical (5 μm) and semi-preparative (5 μm) columns were used. Analytical runs were performed with a 10-70% B gradient over 30 min at 1 mL/min, while semi-preparative runs were optimised for each peptide at 4.7 mL/min. Aqueous CH3CN containing 0.1% TFA was used for buffer A (5%, v/v) and buffer B (95%, v/v). Detection was done at 230 and 214 nm.

### Lipid vesicle preparation

The lipids used for the assembly of unilamellar vesicles were from Avanti Polar Lipids: 1,2-dilauroylphosphatidylcholine (DLPC) was used for zwitterionic vesicles, while its mixture with 1,2-dilauroyl-sn-glycero-3-phospho-(1’-rac-glycerol) (DLPG) at a 3:1 molar ratio was used for anionic vesicles. After weighting up the lipid aliquots were dissolved in chloroform and dried under a nitrogen stream until a thin film was formed. The obtained film was subjected to hydration in 10 mM phosphate buffer (pH 7.4), which was followed by vortexing (1x, 2 min) and sonication (1x, 30 min). A hand-held extruder (Avanti Polar lipids) was used to extrude the resulting suspension (29x, polycarbonate filter, 0.05 μm) until a clear solution containing small unilamellar vesicles (SUV) was obtained. The SUVs were then analysed (50 nm) using a Zetasizer Nano (ZEN3600, Malvern Instruments, UK) and re-suspended to a final concentration of 1 mg/mL. The measurements were performed using a low volume disposable cuvette at 25°C. The manufacture’s software, Dispersion Technology Software (DTS version 5.10), was used to obtain hydrodynamic radii after the fitting of autocorrelation data.

### Circular dichroism spectroscopy

A JASCO J-810 spectropolarimeter was used to record all CD spectra, with measurements taken in millidegrees at 1 nm step, 1 nm bandwidth, 1 second collection time per step and with 4 acquisitions. The obtained spectra were converted to mean residue ellipticities (MRE, deg cm^2^ dmol^-1^ res^-1^) after baseline subtraction and normalisation for the cell pathlength and the concentration of peptide bonds. A quartz cuvette with 0.1 cm pathlength was used for all the measurements. Peptide samples (300 μL, 40 μM total peptide) were prepared in filtered (0.22 μm) aqueous 10 mM phosphate buffer, pH 7.4. Lipid-peptide (L/P) molar ratios of 100 were used for CD spectra that were recorded for peptides in unilamellar phospholipid vesicles.

### Isothermal titration calorimetry

A Microcal isothermal titration calorimeter-200 (ITC-200) was used to carry out all ITC experiments. The calorimeter has a cell volume of ~ 0.2026 mL and a syringe volume of ~ 0.04 mL. The titrations were performed with a 60-s initial delay and a 120-s equilibration time between the start and end of each titration. The experiments were performed with stirring (750 rpm) at 25 °C till no further enthalpy changes could be observed. Binding isotherms were recorded for peptides (500 μM) titrated into unilamellar phospholipid vesicles (380 μM, total lipid) in the cell. Peptide titration into the buffer was used to correct the obtained heats for dilution effects. The proprietary software (Microcal Origin 7.0) was used to analyse the data. One-set binding model was applied to determine association constants (*Ka*) as well as the changes in enthalpy (Δ*H*) and entropy (Δ*S*). Each experiment was done in duplicate.

### Preparation of supported lipid bilayers for in-liquid AFM imaging

A vesicle fusion method was utilized to prepare SLBs as described elsewhere.^9^ Assembled vesicles (5 μL, 1 mg/mL) – DLPC/DLPG (3:1, molar ratio) for anionic SLBs and DLPC for neutral SLBs – were added to cleaved mica pre-hydrated in 20 mM MOPS containing 120 mM NaCl and 20 mM MgCl_2_ (pH 7.4), and incubated over 45 min. After the incubation the samples were washed 10 times with an imaging buffer (20 mM MOPS, 120 mM NaCl, pH 7.4) in order to eliminate unfused vesicles. The obtained SLBs stabilised by divalent cations (Mg^2+^), which bridge negatively charged phosphate headgroups to the negatively charged mica surface, were checked for defects and the bilayer thickness was verified by AFM indentation.^67^ Mica sample discs (Agar Scientific, Stansted, UK) were mounted on steel discs topped with hydrophobic fluorinated ethylene propylene (FEP) coated Bytac laminate (Saint-Gobain, Performance Plastics Corp) to prevent the leakage of aqueous solution during imaging.

### In-liquid AFM imaging of SLBs

Topographic imaging of SLBs – DLPC and DLPC/DLPG (3:1 molar ratio) – was performed in aqueous buffers at room temperature. A Multimode 8 AFM system (Bruker AXS, CA, USA) was used to record images using Peak Force Tapping™ mode and MSNL-E cantilevers (Bruker AFM probes, USA). Images were taken at the PeakForce frequency of 2 kHz, PeakForce amplitude of 10-20 nm and PeakForce set point of 10-30 mV (<100 pN). Image processing was done using Gwyddion (http://gwyddion.net) for flattening (line-by-line background subtraction) and plane fitting. Cross-sections were recorded using Gwyddion and were then plotted using Origin (OriginLab, MA, USA). Peptides diluted in 20 mM MOPS containing 120 mM NaCl (pH 7.4) were transferred into a 100-μL fluid cell (Bruker AXS, USA) to the final concentrations stated.

### Minimum inhibitory concentrations measurements

MIC values were obtained using broth microdilution against different bacteria following the protocols by Clinical and Laboratory Standards Institute. Specifically, each bacterium (100 μL of 0.5–1 × 10^6^ CFU per ml) in Mueller Hinton media broth (Oxoid) was incubated in 96-well microtiter plates at 37 °C on a 3D orbital shaker. 100 μL of serial twofold peptide dilutions (from 100 to 0 μM) were used. Following peptide addition, absorbance (600 nm) was measured in a SpectraMax i3x multi-mode microplate reader (Molecular Devices). The lowest peptide concentrations inhibiting visible bacterial growth after 24 h at 37 °C was defined as MICs. All tests were performed in triplicate. Table S1 summarises the results.

### Hemolysis assay

For hemolysis measurements 10% (vol/vol) suspensions of human erythrocytes with peptides were used. Phosphate buffer saline (PBS, 10 mM, pH 7.2) was used to rinse human red blood cells (4x) using repeated centrifugation and re-suspension (3 min, 3000 × g). The cells were incubated for 1 h at room temperature in deionized water (fully hemolysed control), with peptide in PBS or just PBS, which was followed by centrifugation (5 min, 10000 × g). The obtained supernatant was then separated from the pellet, and absorbance (550 nm) was measured in a SpectraMax i3x multi-mode microplate reader (Molecular Devices). Absorbance obtained for the suspension that was treated with deionized water gave complete (100%) hemolysis. Table S1 summarises the results, with the values corresponding to % hemolysis for peptide at 250 μM. All tests were performed in triplicate.

### Microfluidic assays using anionic GUVs

An octanol-assisted liposome assembly technique was used to prepare GUVs, which were trapped in arrays of hydrodynamic posts and treated with peptides. All the steps were integrated in a single bespoke microfluidic platform. A detailed protocol of the technique, microfluidic designs and GUV assemblies can be found elsewhere.^43, 68^ Anionic GUVs were assembled using the 3:1 mixture (molar ratio) of 1,2-dioleoyl-sn-glycero-3-phosphocholine (DOPC) with 1,2-dioleoyl-sn-glycero-3-phospho-rac-(T-rac-glycerol) sodium salt (DOPG). The lipids were purchased from Avanti Polar Lipids. The GUVs were prepared in sucrose solution (200 mM) with glycerol (15% v/v) in PBS (pH 7.4). The vesicles encapsulate 8-hydroxypyrene-1,3,6-trisulfonic acid (HPTS, 50 μM) – a fluorescent dye purchased from ThermoFisher. The microfluidic assay was controlled by two pressure-driven pump modules (MFCS-4C, Fluigent), and a single neMESYS syringe pump module for fluid manipulation. Once the GUVs were assembled and trapped, the vesicles were perfused continuously overnight (~12 hrs) by the buffer solution used for GUV assembly containing 10 μM of the subject peptide. The perfusion solution contains 5 μM HPTS to mark peptide arrival in the chamber at (t=0). The connected device was mounted onto a motorized Prior XYZ-stage that scans the filled trap arrays, mounted on an Olympus IX73 inverted microscope. The microscope was equipped with a 10× air objective (Olympus UPLFLN) and a wLS LED lamp from QImaging, which was used as the light source for fluorescence imaging. A Photometrics Evolve 512 camera was used to acquire the images of the GUVs. The camera was controlled using μManager 1.4 software which synchronized the acquisition with the automated scanning stage, at a rate of 1 frame per minute for a given ROI. The data was later analysed using a custom Python code that detected the GUVs and collated their fluorescence intensity traces with peptide arrival in the microfluidic chambers. The results are summarised in Figures 3, S9 and S10. The intensity traces are ordered by the critical viability time point. This is the point at which the fluorescence intensity of a vesicle drops below 50% of its initial intensity.

### Bacterial culture for antimicrobial kinetics measurements using single cells

Planktonic bacteria were grown in lysogeny broth (LB) (10 g/L tryptone, 5 g/L yeast extract, 10 g/L NaCl, Melford) and plate bacteria were cultured in LB agar plates (LB with 15 g/L agar). *E. coli* cell line BW25113 was obtained from Dharmacon (GE Healthcare). Single bacterial colonies, picked from a streak plate, were incubated with shaking (200 rpm) in fresh LB (150 mL) over 16 hours at 37 °C. Following incubation, the obtained culture was centrifuged (5 min, 3220 x g) at 20 °C. The supernatant was filtered (2x) using Medical Millex-GS filters (0.22 μm) from Millipore Corp., and then was used to re-suspend the bacterial pellet at the OD_595_ of 75. Single-cell kinetic measurements was performed using the resulting highly concentrated suspension of bacteria, together with M9 minimal medium (1 × M9 salts, 2 mM MgSO_4_, 0.1 mM CaCl_2_, 1 mg/L thiamine hydrochloride) from Sigma Aldrich, and propidium iodide (PI) from Thermo Fisher Scientific. The protocol of the measurements is described below.

### Antimicrobial kinetic measurements using single cells

Aa multi-channel microfluidics device was used to determine the antimicrobial efficacy of the peptides with a single-cell resolution, as described elsewhere.^65^ A polydimethylsiloxane (PDMS, Dow Corning) replica of the original mold, which was kindly provided by Prof Suckjoon Jun (UCSD), was used. This device is an array of microfluidic channels closed at one end with the dimensions of 1.4 μm in height and width and 25 μm in length. A main microchamber (25x100 μm), which is continuously supplied with peptides, fresh LB or propidium iodide (see below), connects the channels. In each channel 1-4 bacterial cells can fit in a single file. Air plasma treatment was used to permanently bind the device to a glass coverslip. The device was then functionalized with bovine serum albumin (50 mg/mL). An aliquot of the highly concentrated *E. coli* suspension (5 μl) was injected into the device to allow for the diffusion of individual bacteria into the channels over 30 min. Fluorinated ethylene propylene inlet and outlet tubing (1/32"×0.008") was used to interface the device with a flow-rate measuring system (Flow Unit S, Fluigent, France). A computerized pressure-based microfluidic flow-control system (MFCS-4C, Fluigent) was used to control the applied pressure. This completed the device, which was then mounted on an inverted microscope (IX73 Olympus, Tokyo, Japan). The microscope was equipped with a 60×, 1.2 N.A. objective (UPLSAPO60XW, Olympus), and a sCMOS camera (Zyla 4.2, Andor, Belfast, UK), which was used to record bright-field images of the cell-hosting channels, with exposure time of 0.03s. M9:LB (9:1 v:v) media containing 10 μM peptide, and flowing at 100 μL/h through the chip over 3 hrs was first used to treat bacteria. Bright-field images were acquired at the initiation of each experiment (t=0) and at hourly intervals thereafter. This was followed by incubation with LB for a further 19 hrs (flow rates of 100 μL/h for the first 3 hrs, during which bright-field images were acquired hourly, and 50 μL/h for the subsequent 16 hrs, overnight). Finally, PI solution (30 μM) was introduced into the device over 15 min, which was used to identify dead cells with compromised cell membranes. Images were acquired in both bright field and epifluorescence (pE-300 white LED light source) to distinguish dead cells and survivors. The microfluidics-microscopy platform enables the tracking of each cell and its progeny throughout the assay. The images were then analysed using FIJI.

### Molecular dynamics

Peptides for bienA and bienK were modelled as ideal helices and initially placed in a configuration parallel to the membrane (Fig 4B). A single peptide was centred on the membrane for PMF calculations; 10 peptides were placed on a grid for classical MD simulations. A 12x12 nm model phospholipid bilayer of the same lipid composition used in the experiments, *i.e*. DLPC/DLPG at 3:1 molar ratio, was used for all the simulations. CHARMM36, CHARMM27 and TIP3P were used for lipids, peptides, and water, respectively, to parameterise peptide-membrane systems. For charge neutralisation sodium counter ions were used.

The following protocol was used to equilibrate all the systems: (i) 5000 minimization steps, followed by (ii) 10 ns with harmonic constraints (1 kcal/mol/A^2^) on protein and lipid heads; (iii) 10 ns with harmonic constraints (1 kcal/mol/A2) on protein only and finally (iv)10 ns without constraints. The classical MD production runs were simulated for 500 ns for each of two independent replicas. Langevin thermostat and Nosé-Hoover Langevin piston barostat were used to keep temperature and pressure constant. The electrostatic energy was evaluated using the particle-mesh Ewald method^69^. A 12 Å cutoff was applied to nonbonded interactions. The NAMD 2.9 software was used for all the simulations.^70^ To estimate the free energy barrier for peptides to translocate the membrane PMF calculations were performed: the adaptive biasing forces (ABF) method was applied as implemented in NAMD. The reaction coordinate was the z-coordinate of the center of mass (COM) of the peptide, relative to the instantaneous midplane of the membrane given by the z-coordinate of the COM of all phosphorus atoms. The calculation was performed starting from the endpoint of the corresponding equilibration phase.

The reaction coordinate was broken down into consecutive windows, and each one of these was simulated for 100 ns. The window size was set to 1 Å. A force constant of 10 (kcal/mol)/Å^2^ was used to limit the sampling in the window. The bin width of 0.1 Å was used for accumulating the instantaneous force. 20 000 samples were set as a threshold before the application of any adaptive biasing force. Different time blocks were used to estimate the standard error in the PMF. Two block sets of 50 ns per window were used to split the calculated data. Spatial coarse-grained models of bienA and bienK (Fig 1D) were built using the Martini framework^71^ and rendered by PyMol. Fractal dimensions of the defects observed for the bienK series were computed using the box counting method with the sliding box algorithm.

## Supplementary Material

Table summarizing biological activities of the peptides and antibiotics used in this study; Figures and a Movie as described in the text including RP-HPLC traces and MALDI-ToF spectra for the peptides; MD simulations for the peptides and their insertion modes in membranes; CD and ITC spectra for peptide folding and thermodynamics in membranes; AFM images of SLBs and microfluidics data for GUVs and live bacteria.

SI movie

Supplementary Information
