# Supplementary material for: Switching Cytolytic Nanopores into Antimicrobial Fractal Ruptures by a Single Side Chain Mutation
Source: ACS Nano. Author manuscript; Available in PMC 2021 Jun 22. (PMC8219408; doi:10.1021/acsnano.1c00218)
Supplement: Supplementary Information [file EMS123265-supplement-Supplementary_Information.pdf]

Supporting Information

Switching Cytolytic Nanopores into Antimicrobial Fractal Ruptures by a Single Side Chain Mutation

Katharine Hammond, Flaviu Cipcigan, Kareem Al Nahas, Valeria Losasso, Helen Lewis, Jehangir Cama, Fausto Martelli, Patrick W Simcock, Marcus Fletcher, Jascindra Ravi, Phillip J Stansfeld, Stefano Pagliara, Bart W Hoogenboom, Ulrich F Keyser, Mark S P Sansom, Jason Crain, and Maxim G Ryadnov

Corresponding author:

Prof Maxim G Ryadnov; National Physical Laboratory,

Hampton Road, Teddington, TW11 0LW, UK

Tel: (+44) 20 89436078; max.ryadnov@npl.co.uk

Table, Figures and Movie

Table S1. Biological activities of peptides used in the study.

cell	bienA			bienK			antimicrobial agents	
	11	10	9	11	10	9	melittin	polymyxin B
	Minimum inhibitory concentration, µM[a]							
E. coli (ATCC 15597)	3	3	6	3	3	3	3	1.5
S. aureus (ATCC 6538)	3	3	25	12	25	12	<1	25
P. aeruginosa (ATCC 27853)	3	25	50	12	25	6	12	1.5
S. typhimurium (6192)	12	6	50	50	50	50	3	1.5
K. pneumoniae (NCTC 5055)	6	3	50	>50	>50	>50	3	3
B. subtilis (ATCC 6633)	6	1.5	6	3	6	3	3	3
M. luteus (ATCC 49732)	1.5	1.5	1.5	1.5	1.5	1.5	<1	1.5
	% hemolysis, at 250 µM							
Human erythrocytes	25	25	25	0	0	0	100	5

[a]shorter sequences showed no activity, >100 µM

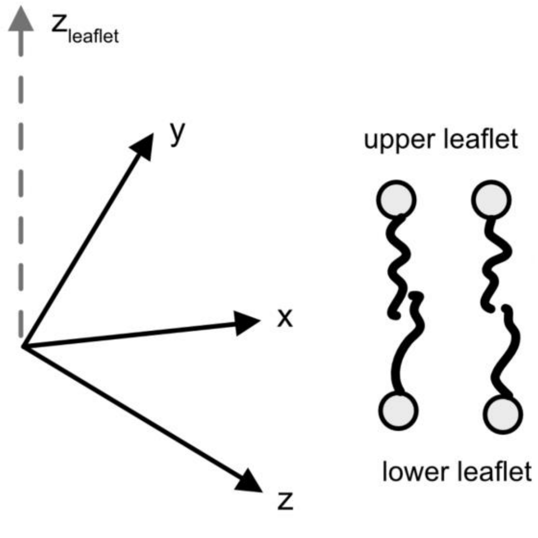

Figure S1. Schematic of the coordinate system used for peptide orientation in phospholipid bilayers.

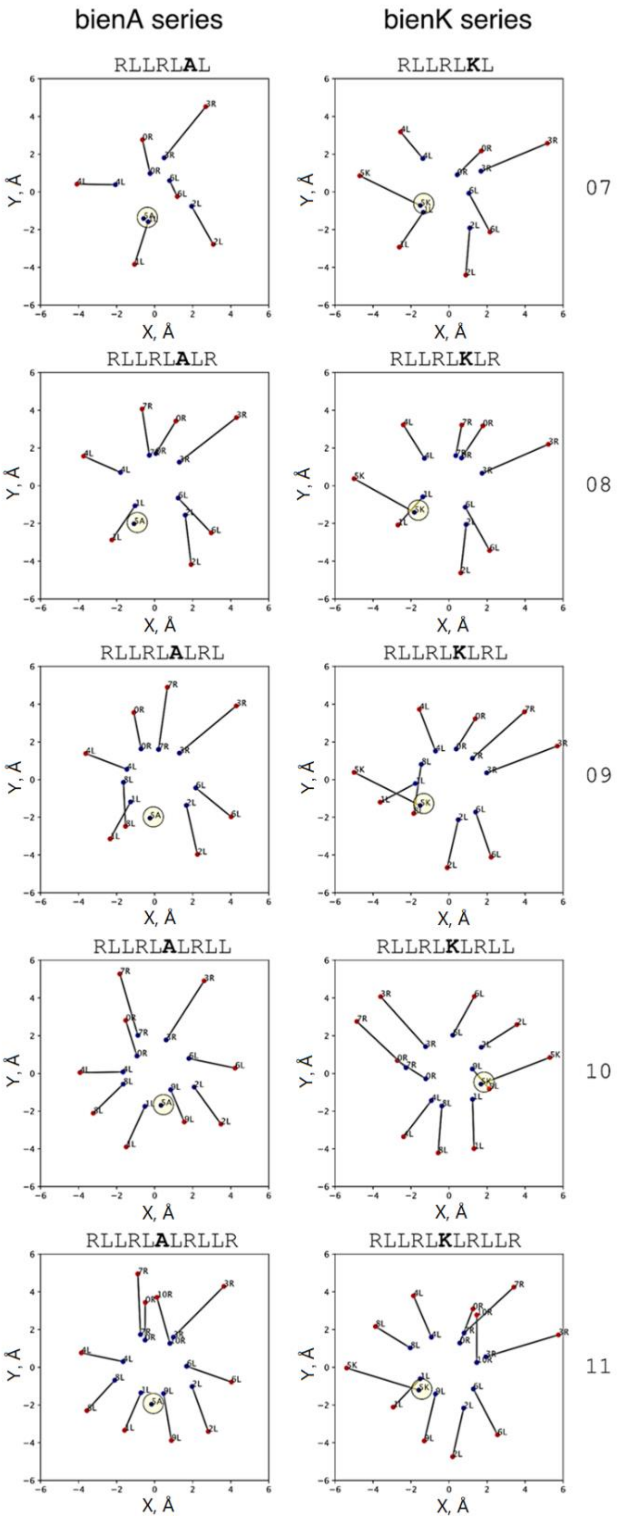

Figure S2. Data driven helical wheels for bienA (left) and bienK (right) computed using the Martini forcefield and methods.[33] The blue circles represent the averaged centre of mass for the backbone of an amino acid, while the red circles denote the centre of mass for its sidechain. The yellow circle highlights the mutation site. Peptides from the bienK series tilt their hydrophilic face due to the reorientation of the Lys residue, resulting in a shallower insertion.

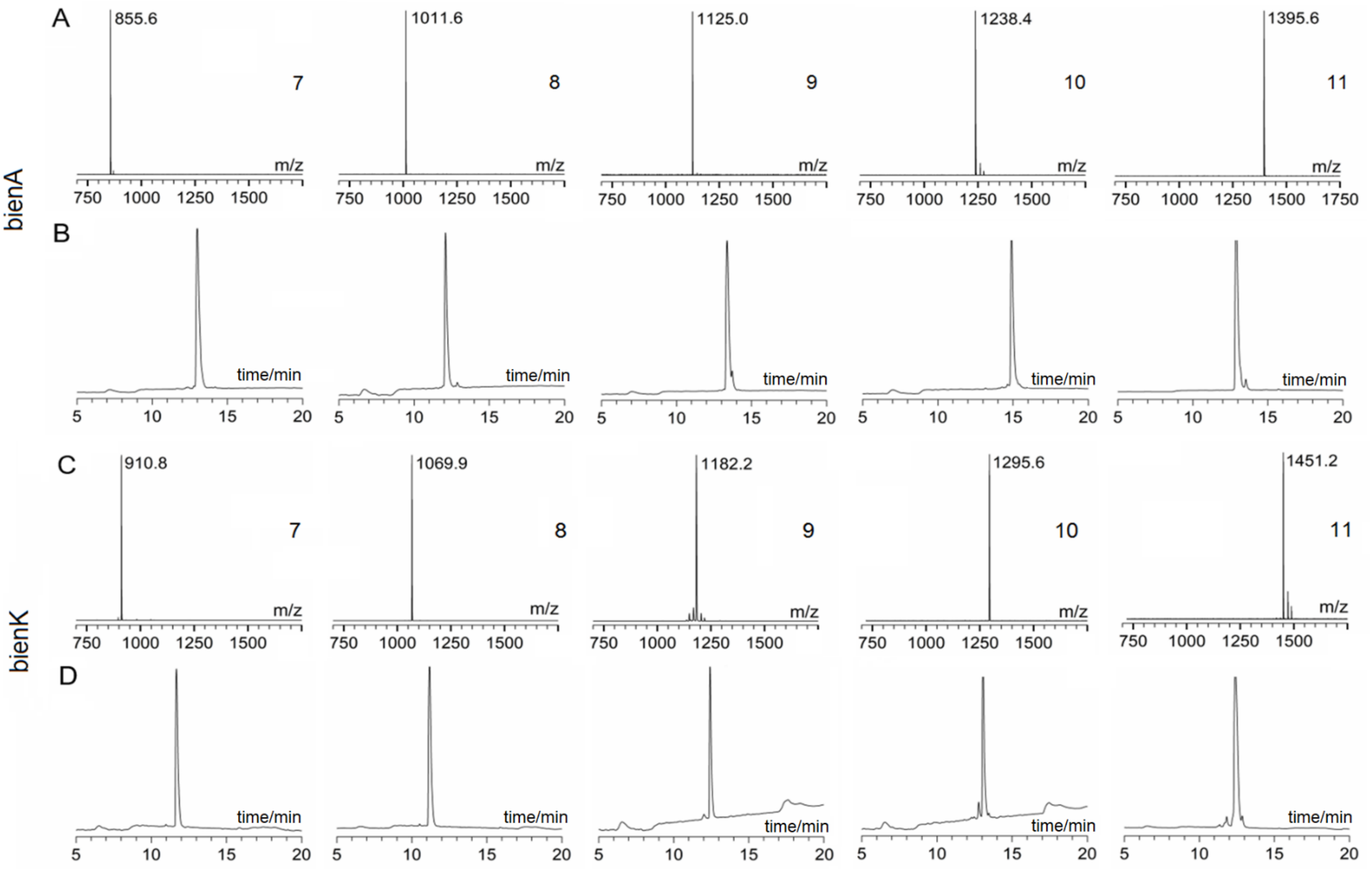

Figure S3. Peptide characterization. MALDI-ToF mass spectra (A and C) and RP-HPLC profiles recorded at 214 nm (B and D) for bienA and bienK peptides.

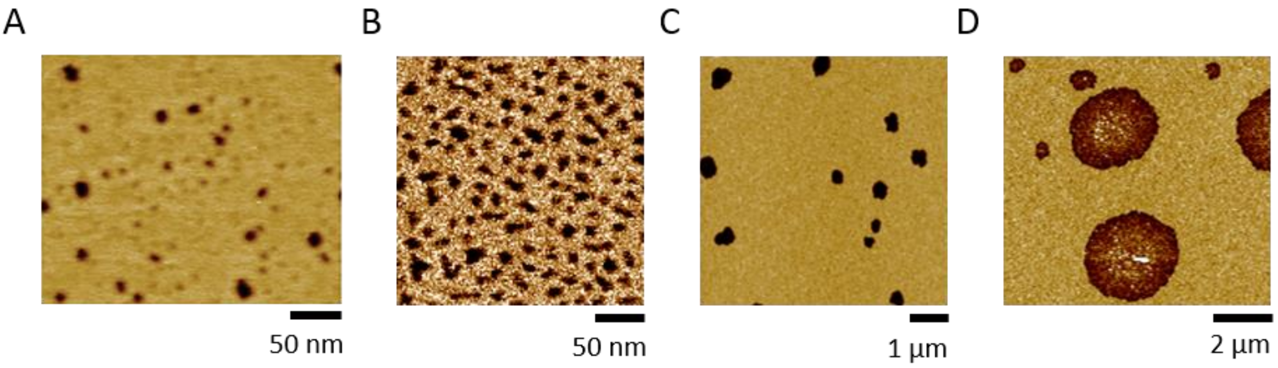

Figure S4. Representative literature examples of peptide-induced circular pores imaged by AFM in supported lipid bilayers. (A) A synthetic peptide forms pH-dependent pores of 2-10 nm in diameter. Reproduced with permission,[28] copyright 2019 American Chemical Society. (B) An archetypal antimicrobial peptide magainin 2, originated from *Xenopus laevis*, forms pores of 10-15 nm in diameter. Reproduced with permission,[21] copyright 2019 American Chemical Society. (C) A human prion peptide, PrP106–126, forms pores hundreds of nanometres in size. Reproduced with permission,[40] copyright 2017 American Chemical Society. (D) A membrane-active protein, α-synuclein, forms micrometre-sized pores. Reproduced with permission under ACS AuthorChoice,[41] copyright 2018 American Chemical Society.

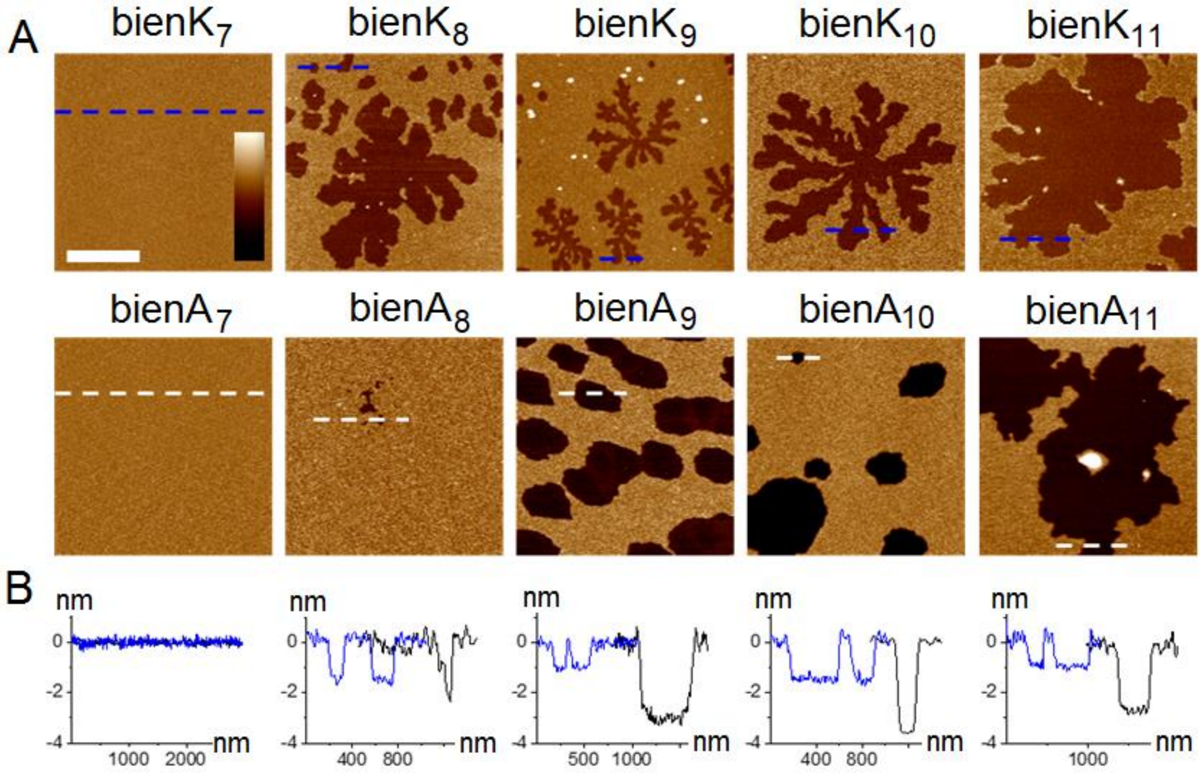

Figure S5. Peptide-induced membrane rupture. (A) In-liquid AFM imaging of SLBs (DLPC/DLPG, 3:1 molar ratio) treated with bienA and bienK peptides (0.3 µM peptide). Length and height scale bars are 1 µm and 7 nm, respectively. (B) Height profiles taken along the highlighted lines in (A) showing the depths of topography defects in the bilayers.

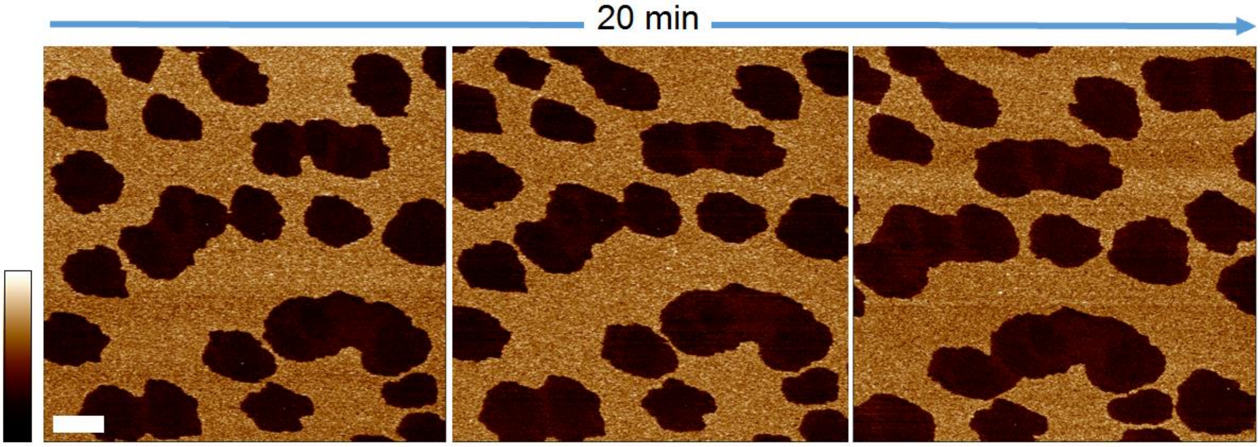

Figure S6. Poration over time. In-liquid AFM imaging of SLBs (DLPC/DLPG, 3:1 molar ratio) treated with bienA_9 (0.3 µM peptide). Length and height scale bars are 500 nm and 7 nm, respectively.

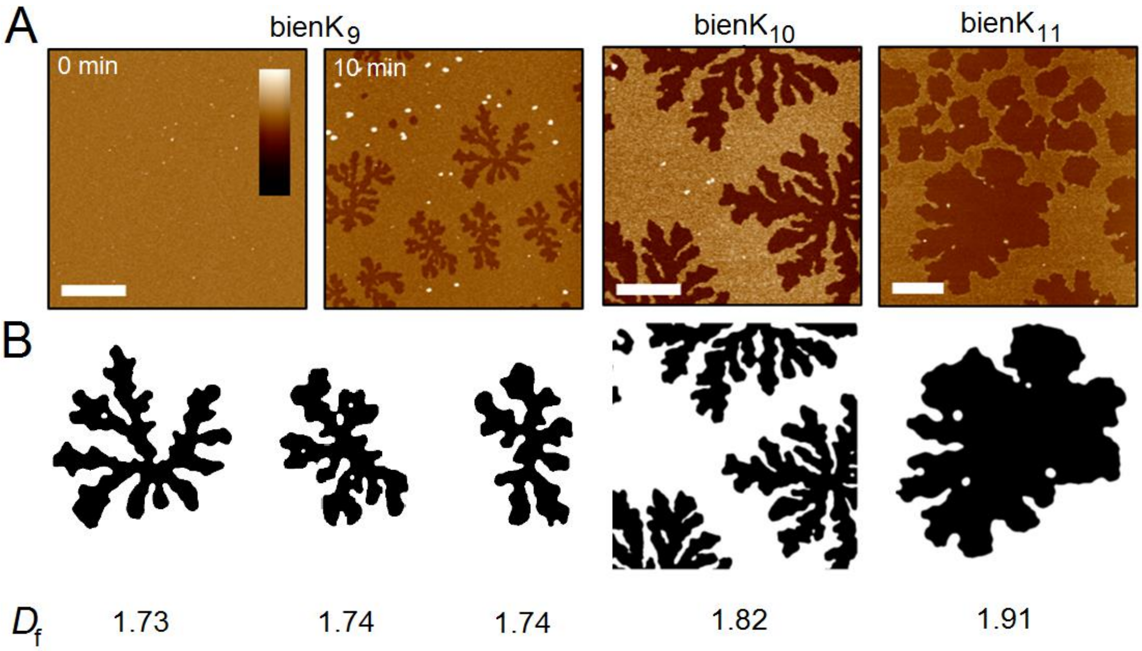

Figure S7. Peptide-induced membrane rupture. (A) In-liquid AFM imaging of SLBs (DLPC/DLPG, 3:1 molar ratio) treated with bienK peptides (0.3 µM peptide). Length and height scale bars are 1 µm and 10 nm, respectively. (B) Fractal contours and calculated dimensions (D_f) for corresponding images.

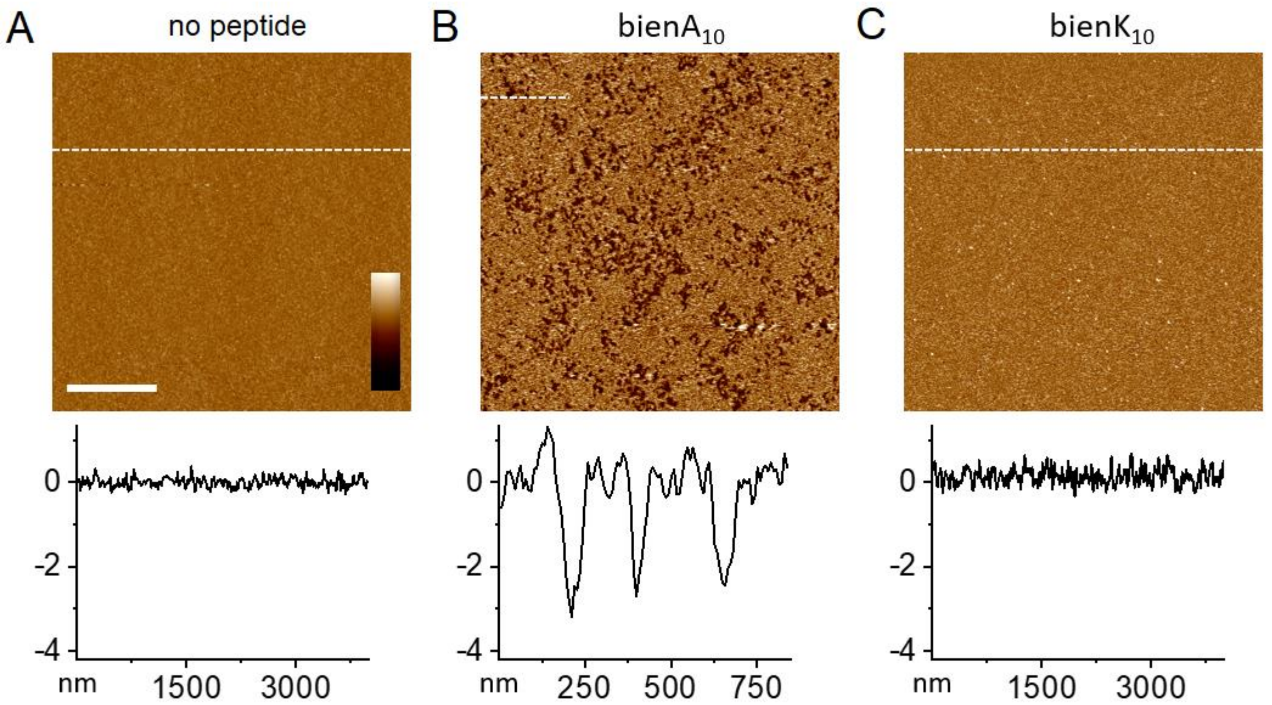

Figure S8. Peptide-treated zwitterionic SLBs. (*Upper panel*) in-liquid AFM imaging of DLPC SLBs without peptide treatment (A) and treated with bienA (B) and bienK (C) at 0.3 µM peptide, over 30 min at room temperature. Length and height scale bars are 1 µm and 8 nm, respectively. (*Lower panel*) height profiles taken along the highlighted lines in (A-C) showing the depth of topography defects in the bilayers.

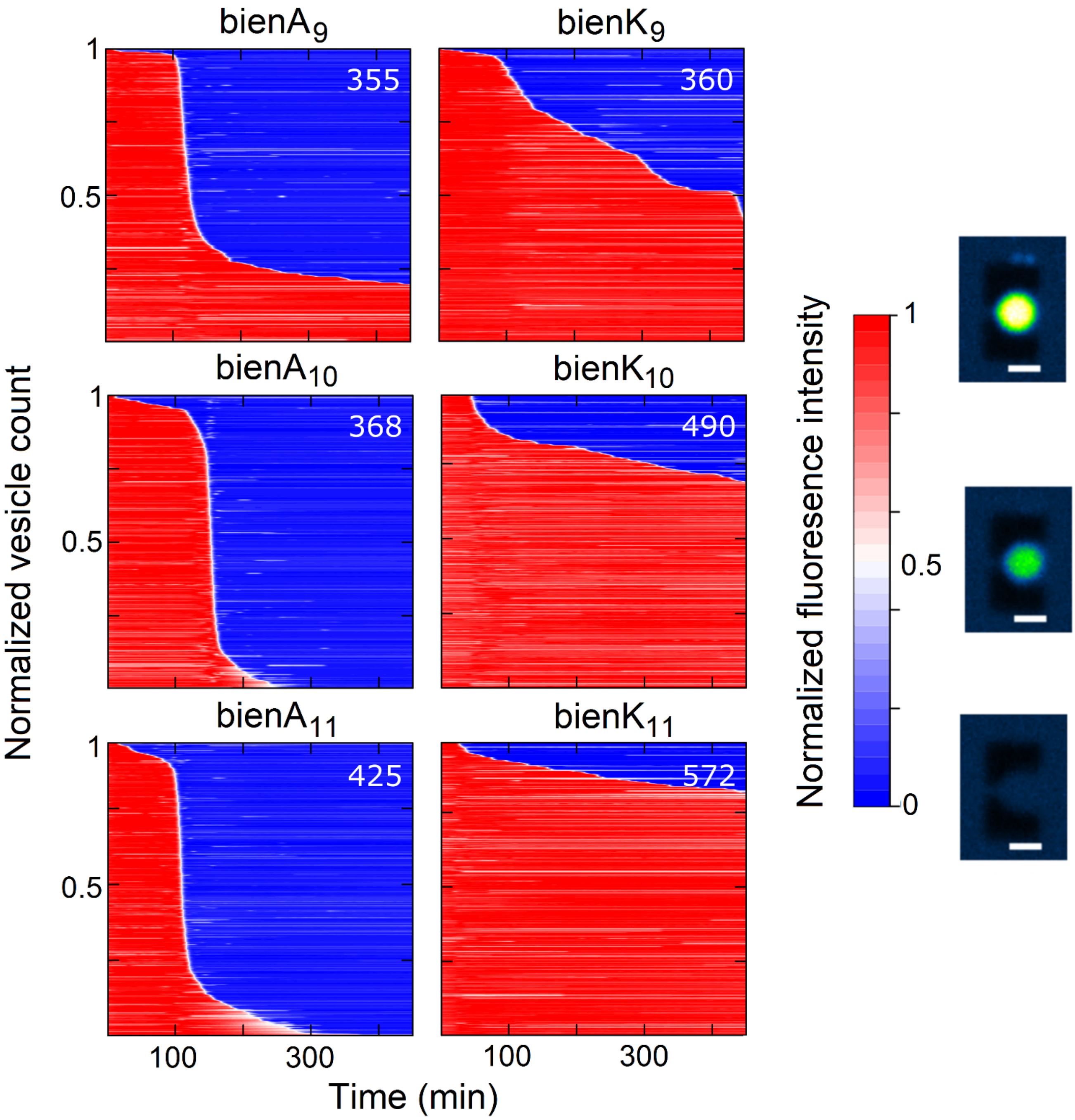

Figure S9. Dye-release membrane rupture of GUVs. Summaries of membranolytic activities for bienA and bienK (10 µM peptide) against GUVs (DOPC/DOPG, 3:1 molar ratio) (left). Each horizontal line in the summaries depicts the locally normalised intensity of an encapsulated dye in a single trapped vesicle over time, after background subtraction. Intact and compromised GUV membranes are at high (red) and low (blue) fluorescence intensities, respectively. Numbers in each summary show the number of analysed vesicles for each case. Fluorescence micrographs of single, trapped GUVs are given along the intensity bar (right). Scale bars are 25 µm.

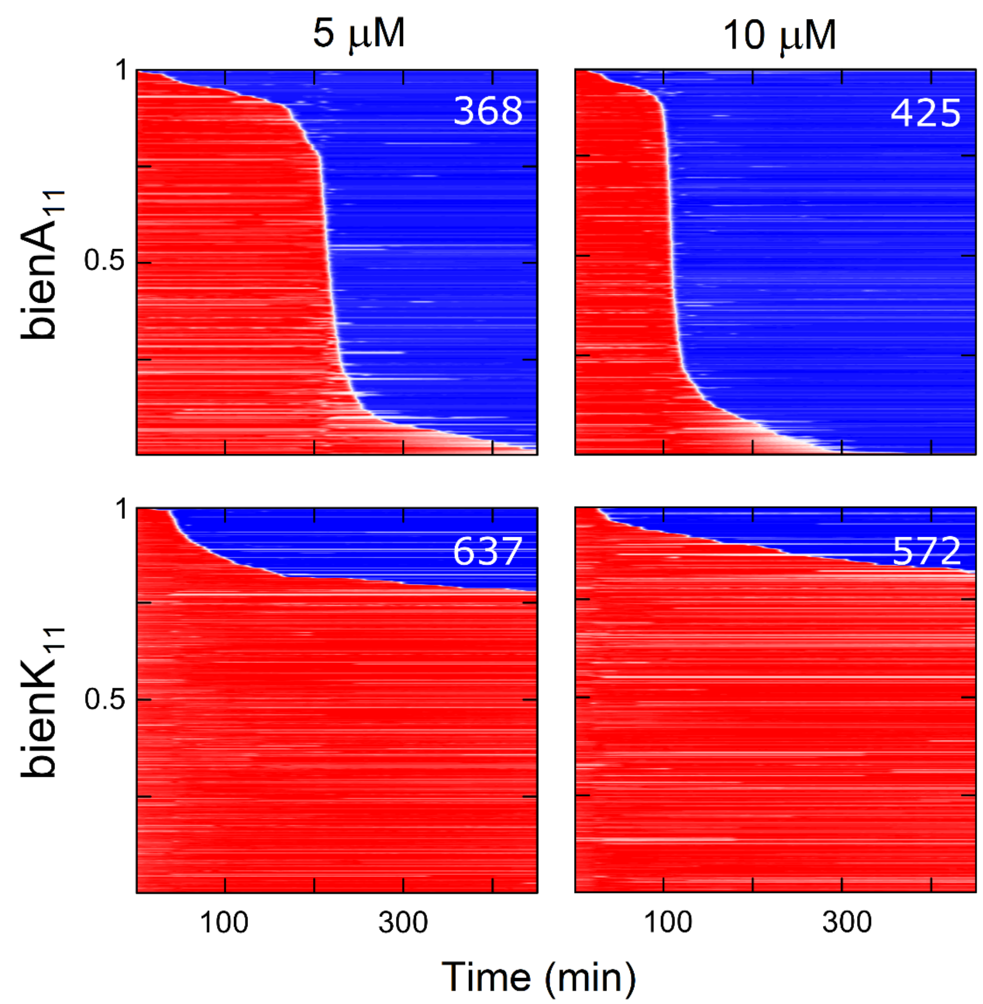

Figure S10. Dye-release membrane rupture of GUVs as a function of peptide concentration. Summaries of membranolytic activities for bienA$_{11}$ and bienK$_{11}$ at 5 μM peptide (left panel) and 10 μM peptide (right panel) against GUVs (DOPC/DOPG, 3:1 molar ratio). Each horizontal line in the summaries depicts the locally normalised intensity of an encapsulated dye in a single trapped vesicle over time, after background subtraction. Intact and compromised GUV membranes are at high (red) and low (blue) fluorescence intensities, respectively. Numbers in each summary show the number of analysed vesicles for each case.

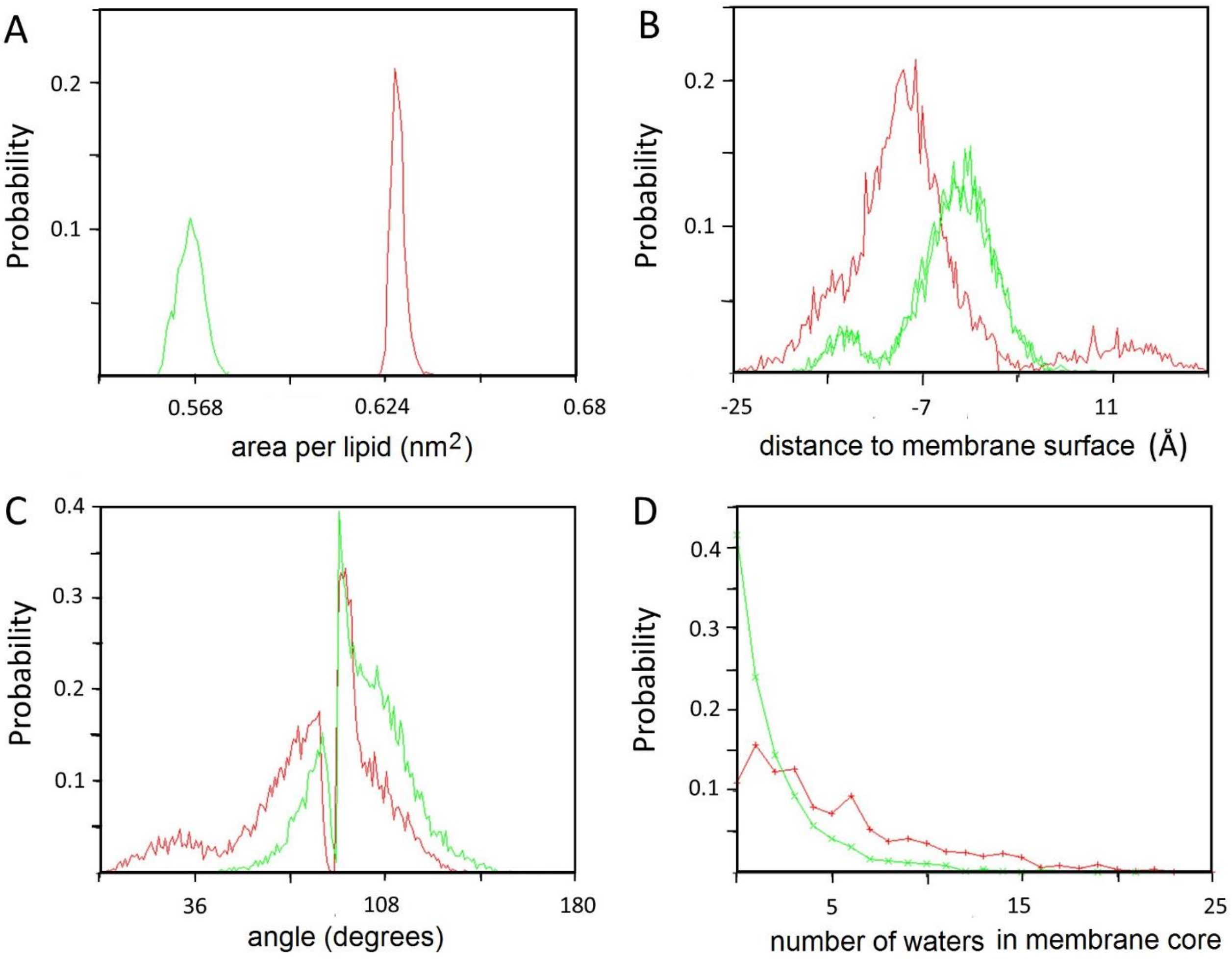

Figure S11. MD simulations of peptide insertion in DLPC/DLPG (3:1 molar ratio). 1-µs simulations of bienA$_9$ and beinK$_9$ run for ten molecules each showing distributions of (A) average area per lipid; (B) distances between peptide centres of mass and the membrane surface; (C) peptide orientations with respect to the membrane normal and (D) distribution of water molecules penetrating the bilayer core (-2.5 Å < z <2.5 Å).

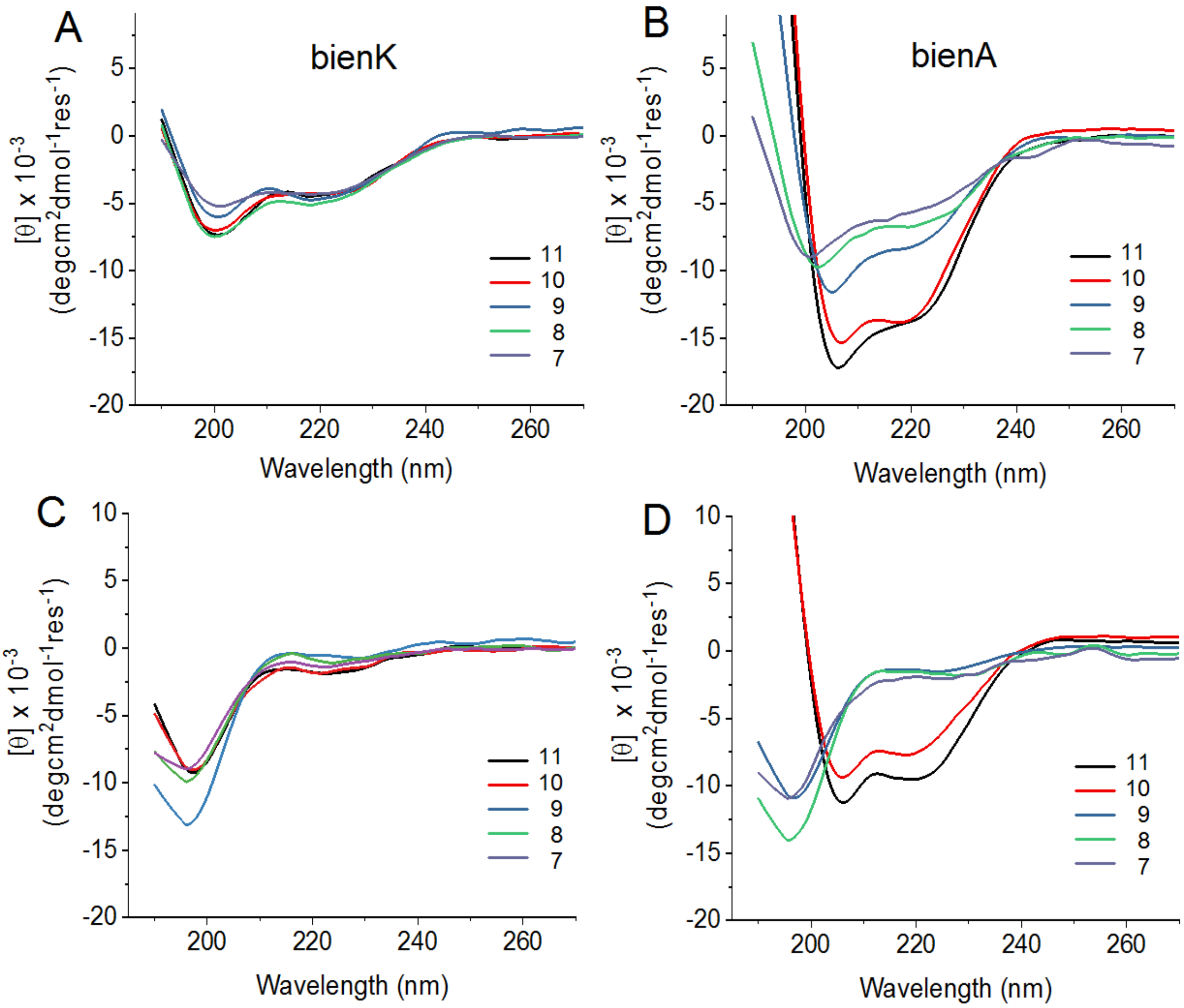

Figure S12. Peptide folding in membranes. Circular dichroism spectra in DLPC/DLPG (3:1 molar ratio) unilamellar vesicles for beinK (A) and bienA (B) and in DLPC unilamellar vesicles for bienK (C) and bienA (D). Key: colour coding is for individual peptides, from 7 to 11-mers, at 40 μM peptide at 100 lipid-to-peptide ratio, in 10 mM phosphate buffer, pH 7.4.

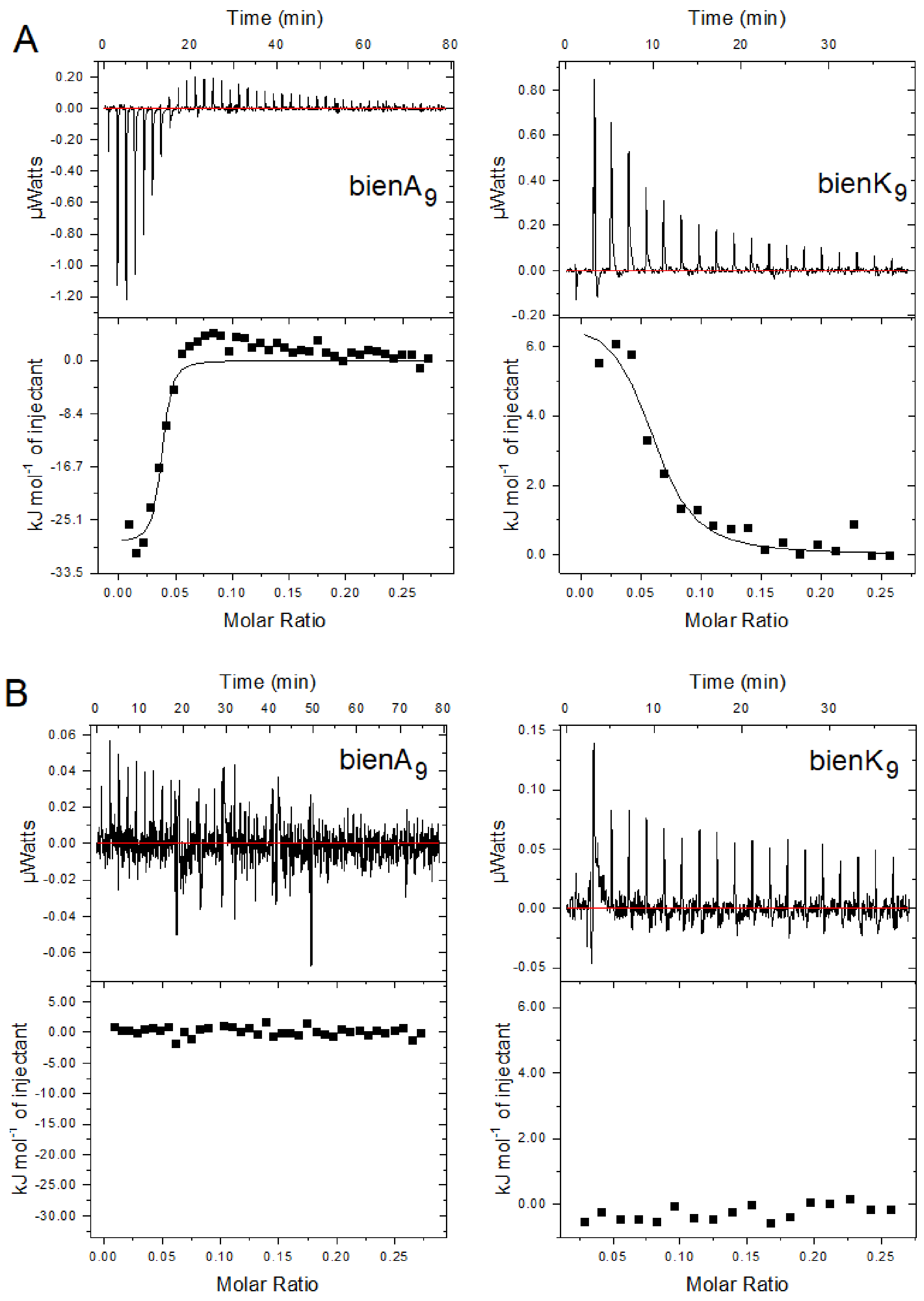

Figure S13. Peptide binding to membranes. Isothermal titration calorimetry of bienA and bienK (500 µM) binding to (A) DLPC/DLPG (3:1 molar ratio) and (B) DLPC unilamellar vesicles. Heat absorbed (μcal/s) for each isotherm is plotted *versus* titration time (upper panel). Integrated heats (kcal/mol) are plotted *versus* protein-lipid molar ratios (lower panel), with a fitting curve shown (black line).

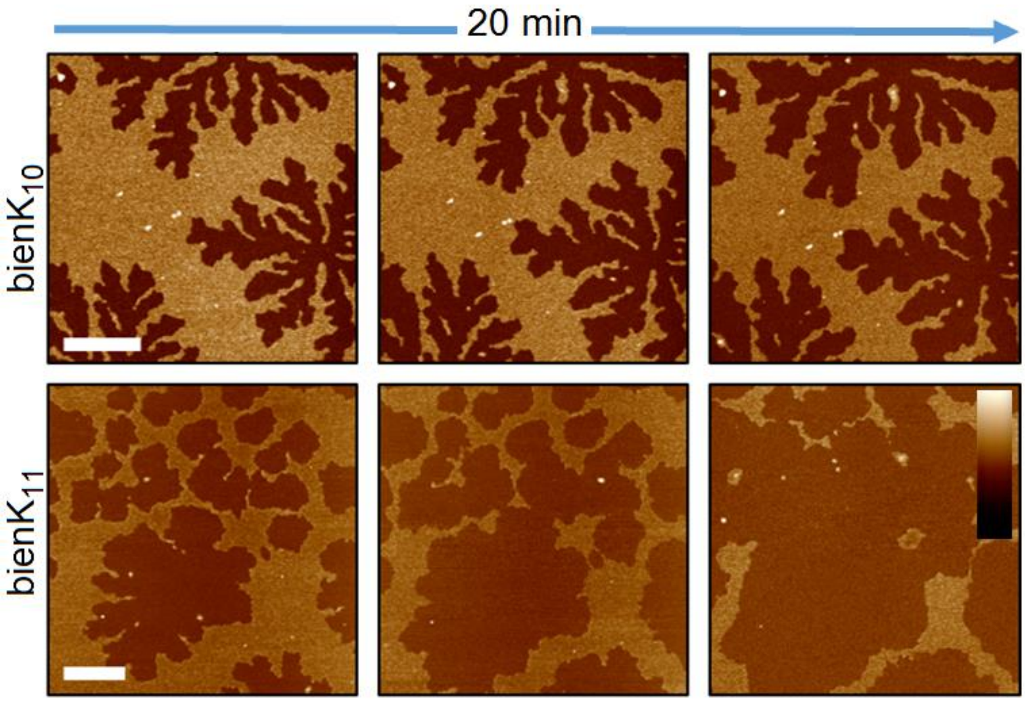

Figure S14. Growth dynamics in fractal ruptures. In-liquid AFM imaging of SLBs (DLPC/DLPG, 3:1 molar ratio) treated with bienK peptides (0.3 µM peptide). Length and height scale bars are 1 µm and 10 nm, respectively.

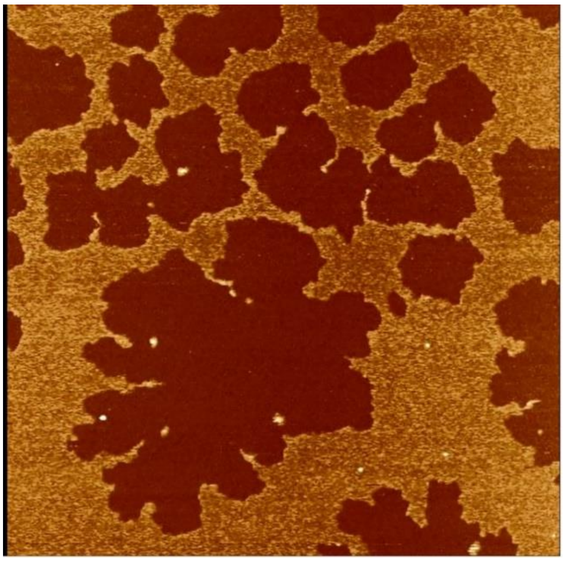

Movie S1. Growth dynamics in fractal ruptures. In-liquid AFM imaging of SLBs (DLPC/DLPG, 3:1 molar ratio) treated with bienK_{11} (0.3 µM peptide). The video is a sequence of time-lapse images (5 μm^2 scans) recorded over 1 hour at 8 min per image: a snapshot of the video is shown.
